# Early Prediction of Cognitive Deficit in Very Preterm Infants Using Brain Structural Connectome With Transfer Learning Enhanced Deep Convolutional Neural Networks

**DOI:** 10.3389/fnins.2020.00858

**Published:** 2020-09-18

**Authors:** Ming Chen, Hailong Li, Jinghua Wang, Weihong Yuan, Mekbib Altaye, Nehal A. Parikh, Lili He

**Affiliations:** ^1^The Perinatal Institute, Cincinnati Children’s Hospital Medical Center, Cincinnati, OH, United States; ^2^Department of Electronic Engineering and Computing Systems, University of Cincinnati, Cincinnati, OH, United States; ^3^Department of Radiology, University of Cincinnati College of Medicine, Cincinnati, OH, United States; ^4^Department of Radiology, Cincinnati Children’s Hospital Medical Center, Cincinnati, OH, United States; ^5^Division of Biostatistics and Epidemiology, Cincinnati Children’s Hospital Medical Center, Cincinnati, OH, United States; ^6^Department of Pediatrics, University of Cincinnati College of Medicine, Cincinnati, OH, United States

**Keywords:** convolutional neural network, deep learning, cognitive deficit, transfer learning, structural connectome

## Abstract

Up to 40% of very preterm infants (≤32 weeks’ gestational age) were identified with a cognitive deficit at 2 years of age. Yet, accurate clinical diagnosis of cognitive deficit cannot be made until early childhood around 3–5 years of age. Recently, brain structural connectome that was constructed by advanced diffusion tensor imaging (DTI) technique has been playing an important role in understanding human cognitive functions. However, available annotated neuroimaging datasets with clinical and outcome information are usually limited and expensive to enlarge in the very preterm infants’ studies. These challenges hinder the development of neonatal prognostic tools for early prediction of cognitive deficit in very preterm infants. In this study, we considered the brain structural connectome as a 2D image and applied established deep convolutional neural networks to learn the spatial and topological information of the brain connectome. Furthermore, the transfer learning technique was utilized to mitigate the issue of insufficient training data. As such, we developed a transfer learning enhanced convolutional neural network (TL-CNN) model for early prediction of cognitive assessment at 2 years of age in very preterm infants using brain structural connectome. A total of 110 very preterm infants were enrolled in this work. Brain structural connectome was constructed using DTI images scanned at term-equivalent age. Bayley III cognitive assessments were conducted at 2 years of corrected age. We applied the proposed model to both cognitive deficit classification and continuous cognitive score prediction tasks. The results demonstrated that TL-CNN achieved improved performance compared to multiple peer models. Finally, we identified the brain regions most discriminative to the cognitive deficit. The results suggest that deep learning models may facilitate early prediction of later neurodevelopmental outcomes in very preterm infants at term-equivalent age.

## Introduction

A high prevalence of long-term cognitive deficit is well-established in very preterm infants (≤32 weeks’ gestational age), with 35–40% of this population identified with a deficit at 2 years of age (Blencowe et al., 2012; Hamilton et al., 2016). This neurological deficit may affect the infant throughout life, thereby resulting in difficulties in academic skills and building social relationships. Yet, no robust prognostic screening technique is available following neonatal intensive care stay. Typically, an accurate diagnosis of cognitive deficit cannot be made until early childhood around 3–5 years of age. This delayed diagnosis misses the optimal neuroplasticity period of brain development in the first 3 years of life and potentially undermines the effectiveness of early interventions. As such, reproducible approaches that serve as neonatal prognostic tools are needed to fill the gap in our knowledge about the early prediction of cognitive deficit in very preterm infants.

The human brain is a highly interconnected network with coordinated information transfer among individual brain regions (Sporns et al., 2005). Advanced non-invasive neuroimaging MRI techniques have been applied to construct such network representation of the brain, referred to as the brain connectome (Bullmore and Sporns, 2009). Theoretically, a brain connectome is a graph, where vertices represent a set of brain regions of interest (ROIs) and edges represent brain connectivity between ROIs. This brain connectome perspective shifted traditional research that focuses on isolated ROIs toward research on a systematic mechanism incorporating the whole brain. Brain connectome data have very high dimensionality and are intrinsically complex, creating difficulties in designing feature extraction methods and building analysis models. Deep learning has shown great promise in deciphering complex and high dimensional data (e.g., images, signals, and videos) to achieve superior performance in numerous fields, including computer vision, speech recognition, and natural language processing (LeCun et al., 2015). Indeed, numerous studies have applied deep learning approaches to brain connectome for various neurological disorders (Wee et al., 2012; Barkhof et al., 2014; He et al., 2018; Heinsfeld et al., 2018; Li et al., 2018; Sen et al., 2018; Chen et al., 2019).

Brain connectome plays an important role in understanding human cognitive functions (Nagy et al., 2004; Park and Friston, 2013; Petersen and Sporns, 2015). Recent research demonstrated that deep learning models were capable of predicting later cognitive deficits for neonates using brain structural connectome that was constructed by diffusion tensor imaging (DTI) data (Kawahara et al., 2017; Girault et al., 2019). One method to apply deep learning models to brain connectome data is to ignore the topology of the connectome and reshape the adjacency matrix into a vector of features as input (Munsell et al., 2015; Girault et al., 2019). However, the spatial locality (i.e., 2D grid regions of an adjacency matrix) and topological locality information (i.e., rows/columns of an adjacency matrix) in the brain connectome are not utilized, thereby resulting in information loss and potentially compromising the performance of prediction models. Another approach is to apply specialized topological row and column filters on the adjacency matrix of the brain structural connectome to learn the topological relationship between edges (Kawahara et al., 2017). This approach, however, only emphasizes topological locality and discards the spatial locality information (e.g., physically nearby brain ROIs and associated edges) that are intrinsic to any brain ROI parcellation. Since the brain structural connectome is a modular graph that contains clusters of vertices and edges, its adjacency matrix contains hierarchically segregated modules (Park and Friston, 2013). Those topological filters may extract redundant information within connectome modules and may not be efficient for capturing spatial locality. In this work, we consider the adjacency matrix of brain structural connectome as a 2D image and propose to apply established deep convolutional neural networks (CNNs) to learn the spatial and topological information of the brain connectome.

Although deep CNN models have shown promising results on image classification, those models usually require large datasets for model training. In the studies of very preterm infants, available annotated neuroimaging datasets with clinical and outcome information are usually limited and expensive to enlarge, preventing deep CNN to be directly utilized. Transfer learning (TL) may serve as a potential solution to this challenge. Briefly, TL reuses a pre-trained model designed for one task as a starting point for another related task (Bengio, 2012; Samala et al., 2016, 2018; Shin et al., 2016; Azizi et al., 2017; Kooi et al., 2017; Zheng et al., 2018; Bizzego et al., 2019). Raina et al. (2007) proposed a self-taught learning framework that takes unlabeled images to improve the classification performance of their target classification task. Cheng et al. (2019) transferred image features learned from the early stages of Alzheimer’s disease (AD) to improve the prediction of AD diagnosis. Gao et al. (2019) reused pre-trained models based on a large-scale natural image dataset and re-trained a deep learning model for classification of brain activity heatmaps derived from task-based functional MRI data. Recently, we applied the TL technique to a deep neural network (DNN) model for cognitive deficit prediction using brain functional connectome data (He et al., 2018). The DNN model was pre-trained using a large number of brain connectome data in an unsupervised fashion and then fine-tuned with brain connectome data from very preterm infants.

In this study, we proposed a TL-enhanced deep CNN (TL-CNN) model for early prediction of cognitive deficit at 2 years of age in very preterm infants using brain structural connectome derived from at term DTI data. Specifically, the proposed model contains two modules, a very deep CNN (which was trained with supervision using ∼1.2 million images from the ImageNet database) (Deng et al., 2009) and a “shallow” CNN. With the fixed weighted pre-trained very deep CNN, we only need to train and fine-tune the “shallow” CNN using available very preterm infants’ brain connectome data and associated risks of cognitive deficit. For individual very preterm infants, we constructed brain structural connectome using mean fractional anisotropy from their DTI data collected at term-equivalent age. The proposed model is able to evaluate at term whether or not a very preterm infant will have a high risk to develop later cognitive deficits as well as to predict this infant’s cognitive assessment [standardized Bayley Scales of Infant and Toddler Development, Third Edition (Bayley III) cognitive score] at 2 years of age.

## Materials and Methods

### Subjects

The study includes a cohort of 110 very preterm infants, born at 31 weeks gestational age or less from four academic and non-academic centers in Columbus, Ohio, including Nationwide Children’s Hospital (NCH), Ohio State University Medical Center, Riverside Hospital, and Mount Carmel St. Ann’s Hospital. Infants were enrolled between December 2014 and April 2016. All subjects with any congenital or chromosomal anomalies affecting the central nervous system were excluded. Infants with cyanotic congenital heart disease were also excluded. The study was approved by the Institutional Review Board of NCH. Approval at the other hospitals was obtained through reciprocity agreements that were in place with NCH. Written informed consent was obtained from parents or legal guardians of all infants.

### MRI and Cognitive Outcome Acquisition

Very preterm infants in the cohort were scanned on a 3T scanner (Skyra; Siemens Healthcare) at NCH using a 32-channel phased-array head coil. The imaging was performed after the infant was fed and in natural sleep without sedation. Natus Mini Muffs (Natus Medical Inc., Scan Carlos, CA, United States) and InstaPuffy Silicone Earplugs (E.A.R Inc., Boulder, CO, United States) were employed for MRI noise reduction. DTI was acquired with echo-planar imaging using the following parameters (b800/b2000): repetition time = 6972/5073 ms; echo time = 88 ms; field of view = 160 mm × 160 mm; in-plane resolution = 2 mm × 2 mm; number of slices = 76; slice thickness = 1 mm; 64 non-colinear diffusion-weighted directions; for all images, one volume has no diffusion sensitization; sensitivity encoding factor equates to 2. High-resolution T2-weighted anatomical images were acquired with rapid spin-echo sequence: TR/TE = 7.3/3.4 ms, flip angle = 11°, voxel dimensions 1.0 mm × 1.0 mm × 1.0 mm, scan time = 2:47 min.

All preterm infants received (Bayley-III) test at 2 years corrected age while blinded to DTI data. The Bayley-III cognitive scores are on a scale of 40–160, with a mean of 100 and a standard deviation of 15.

### DTI Data Preprocessing

DTI data were preprocessed using FMRIB’s Diffusion Toolbox (in the FMRIB Software Library, FSL, Oxford, United Kingdom) following our previously established pipeline (Yuan et al., 2015). Specifically, head motion and eddy current artifacts were mitigated by aligning all diffusion images to the b0 image via an affine transformation. Diffusion tensor reconstruction and brain fiber tracking were performed in the subject’s native space using Diffusion Toolkit/TrackVis (Hess et al., 2006; Wang et al., 2007). Diffusion tensor calculation was based on a linear least-square fitting algorithm, and brain fiber tracking was based on a deterministic tracking algorithm (Wang et al., 2007). The fiber tracking uses an angular threshold of 35°. The fiber length threshold was set to 5 mm. The obtained fractional anisotropy maps were harmonized using a batch-effect correction algorithm ComBat (Fortin et al., 2017). We use a neonatal Automated Anatomical Labeling (AAL) brain atlas proposed by Shi et al. (2011). For each subject, the high-resolution T2-weighted images were first registered to the b0 image in the subject’s native space and then to the neonatal template space to obtain a transformation matrix. Next, the inverse transformation matrix was used to transform the parcellated ROIs from the template space back to the subject’s native space (b0).

### Whole-Brain Structural Connectome Construction

A brain connectome is a graph *G* = (*A*,Ω), where vertices Ω represent a set of ROIs, and *A* is an adjacency matrix of edges that represent brain connectivity between a pair of ROIs. Ninety ROIs were defined based on a neonatal automated labeling atlas (Shi et al., 2011). The weights of structural connectivity between each pair of ROIs were calculated as the mean FA of all voxels along the WM tract constructed between the two ROIs, resulting in a 90 × 90 symmetric adjacency matrix. This was performed using the UCLA Multimodal Connectivity Package (Bassett et al., 2011).

### Overview of TL-Enhanced Deep CNN

**FIGURE 1:**
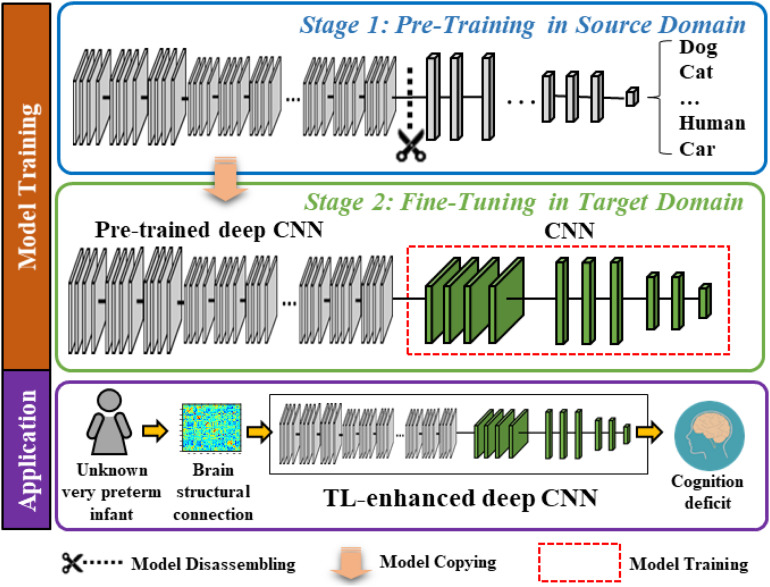
Schematic diagram of the proposed transfer learning-enhanced deep CNN (TL-CNN) model to predict cognitive deficits at 2 years corrected age using brain structural connectome data obtained at term in very preterm infants. The top two blocks demonstrate a two-stage model training procedure, and the bottom block illustrates a potential clinical computer-aided diagnosis application after model training.

The proposed model contains two modules, a very deep CNN (which was trained with supervision using ∼1.2 million images from the ImageNet database) (Deng et al., 2009) and a “shallow” CNN. In Figure 1, we display a two-stage model training procedure in the top two blocks and picture a clinical application in the bottom block, where the proposed model can aid clinicians in the prediction of cognitive deficit using brain structural connectome data. The model training procedure contains two stages: (1) pre-training in the source domain and (2) fine-tuning in the target domain. Specifically, in stage 1, we first pre-trained a deep CNN to learn the basic transferrable image representation (e.g., edges, shapes, etc.) using a large number of color images and associated image labels (source domain). In stage 2, we reused the pre-trained model from stage 1 and fine-tuned the model in the target domain with brain structural connectome and associated cognition deficit outcomes.

### Pre-training in the Source Domain

In the source domain, we trained the proposed model to learn transferrable image representation (e.g., edges, shape, and blobs) from diverse objects (e.g., animals, vehicles, human, and natural environments). We defined the task in the source domain as an image classification task. Adjacency matrices of brain connectome are different from those semantic images (dogs, cats, etc.); however, the low-level imaging features (for example straight and curved lines that construct images) are universal to most image analysis tasks. Therefore, the idea behind TL is to treat the pre-trained model as a feature extractor to extract low-level imaging features from the adjacency matrix of a given structural connectome. In this study, we started with the VGG-Nets (Simonyan and Zisserman, 2014) to develop our deep CNN model. VGG-Nets are a set of very deep CNN that were initially proposed by Visual Geometry Group in ImageNet Large Scale Visual Recognition Competition (ILSVRC) 2014. They have been applied to other image analysis applications (Choi et al., 2017; Zhen et al., 2017; Wang et al., 2018). We adopted the architecture of VGG19, one of VGG-Nets models for our study. Briefly, VGG19 is a very deep CNN that consists of 19 trainable layers, including 16 convolutional layers and 3 fully connected (FC) layers designed for classifying 1000 object categories. For each convolutional layer, the VGG19 uses small convolutional filters (3 × 3) along with a rectified linear unit activation function. We obtained a VGG19 model that was pre-trained using ∼1.2 million color images from the ImageNet database. We then dissembled the model and reserved the weights of the convolutional and pooling layers (Figure 1, blue box).

### Fine-Tuning in the Target Domain

The task in the target domain is to predict the cognitive outcome at 2 years corrected age using brain structural connectome obtained at term-equivalent age. Since the deep CNN in the source domain was pre-trained to recognize transferrable image representation, it would automatically extract image features from the brain structural connectome. The fine-tuning in the target domain is essential to discover discriminative features among generic features and link them to the target task (i.e., cognitive development). We connected a “shallow” CNN (i.e., 2 convolutional layers and 2 FC layers) to the pre-trained fixed weighted deep CNN from the first stage. Finally, an output layer was attached for classification or regression tasks (Figure 1, green box). We used brain structural connectome and follow-up cognitive outcomes to fine-tune the deep CNN model. Given *N* training samples (**x**_1_,**x**_2_,…,**x**_*i*_,…,**x**_*N*−1_,**x**_*N*_) from the target cohort as well as their labels (*y*_1_,*y*_2_, …,*y*_*i*_,…,*y*_*N*−1_,*y*_*N*_), where **x**_*i*_ is the *i*-th input sample (i.e., brain structural connectome) and *y*_*i*_ is the corresponding label, we defined the cross-entropy loss function as:

$$J\,\left(\text{W},b\right)=-\frac{1}{N}\,\sum_{i=1}^{N}y_{i}\,\log\left(p\,\left({\text{x}}_{i}\right)\right)+\left(1-y_{i}\right)\,\log\left(1-p\,\left({\text{x}}_{i}\right)\right)$$

where *p*(**x**_**i**_) is the predicted probability of **x**_**i**_, W is the weight matrix, and ***b*** denotes the bias of the model. In addition to the dichotomized prediction (i.e., classification), we also trained our model to perform continuous cognitive score prediction (i.e., regression). We applied a linear unit at the end of the model and optimized a weighted mean absolute error (MAE) loss function as follows:

$$L\,\left(W,b\right)=\frac{1}{N}\,\sum_{i=1}^{N}\left|\left(y_{i}-{\hat{y}}_{i}\,\left(\text{W},b\right)\right)\right|$$

where ${\hat{y}}_{i}\,\left(W,b\right)$ is the output of the linear unit of the model, i.e., the predicted score. Similar to the previous cross-entropy loss function, **b** represents the bias, and *W* is the weight of the model. The proposed model was optimized using Adam (Kingma and Ba, 2014), a backpropagation gradient descent algorithm. Adam computes adaptive learning rates for weight updating based on the average of recent magnitudes of the gradients, improving computational efficiency. The initial learning rate is set to 0.001. We applied 50 epochs to train the TL-CNN model. The detailed architecture of the TL-CNN model is elaborated in Supplementary Figure 1.

### Alternative Model Comparison

#### Linear/Logistic Regression Model

In the linear regression model, we applied mean squared error as the loss function to minimize the residual sum of error between the true score and the score predicted by the linear approximation. For the logistic regression (LR) model, we adopted cross-entropy as the cost function. We used L2 regularization as the penalty term, and we grid searched the regularization parameters with empirical values (10^–3^, 10^–2^,…, 10^1^).

#### Support Vector Machine

We tested the support vector machine (SVM) model with three different kernels: linear, polynomial, and radial basis function (RBF), where the SVM with linear kernel achieved the best prediction performance. Specifically, for all SVM models, we used L2 regularization as the penalty. We grid searched the regularization parameters with empirical values (10^–3^, 10^–2^,…, 10^1^) and the soft margin parameter C with empirical values (2^–3^, 2^–2^,…, 2^3^) to optimize the prediction performance. For the polynomial and RBF SVM model, we set the scale gamma kernel coefficient as 1.

#### Deep Neural Network

The DNN model has an input layer, two FC layers with 256 and 64 neurons, and an output layer. The rectifier linear unit as activation function was used in each neuron. We attached a batch normalization layer and a dropout layer after each FC layer. In the output layer, we used a SoftMax classifier for classification and a linear classifier for regression. The DNN was trained in a supervised fashion and tested using the labeled subjects from the target domain.

#### TL-DNN

The TL-DNN model has the same structure as the DNN model. Instead of training from scratch, we pre-trained the TL-DNN model in an unsupervised fashion using 257 full-termed neonatal subjects from the source domain. Then, we fine-tuned the TL-DNN with supervision using the labeled subjects from the target domain.

#### Convolutional Neural Network

The CNN model has two convolutional layers, where each has 256 neurons with a 3 × 3 convolutional filter, and two FC layers, where each layer contains 256 and 64 neurons. A rectified linear unit was used as an activation function. A batch normalization and a dropout layer are attached after each FC layer. We applied a SoftMax classifier for the classification task and linear function for the regression task. The architecture design of this model represents a standard “shallow” CNN model without TL strategy. The CNN model was trained and tested using the subjects from the target domain.

### Data Augmentation

The number of very preterm infants in the study cohort is relatively small and imbalanced (i.e., only a small portion of the cohort are at high risk for cognitive deficit). We utilized the synthetic minority over-sampling technique (SMOTE) (Chawla et al., 2002) to balance and augment the training set. Specifically, the training subjects were divided into five bins according to their scores (<70, 70–80, 80–90, 90–100, and >100). Given a bin, a sample was randomly chosen. Then, *k* nearest neighbors for the selected sample were searched. We set *k* = 5 in this work. A synthetic sample *x*_*syn*_ is calculated using the randomly selected sample and its associated neighbors *x*_*1*_, *x*_*2*_, *x*_*3*_, *x*_*4*_, *x*_*5*_, *x*_*6*_ by: *x*_*syn*_=*w*_1_*x*_1_ + *w*_2_*x*_2_ + *w*_3_*x*_3_ + *w*_4_*x*_4_ + *w*_5_*x*_5_ + *w*_6_*x*_6_, where *w*_*1*_, *w*_*2*_, *w*_*3*_, *w*_*4*_, *w*_*5*_, and *w*_*6*_ are random numbers and *w*_1_ + *w*_2_ + *w*_3_ + *w*_4_ + *w*_5_ + *w*_6_ = 1. Similarly, the label *y*_*syn*_ for *x*_*syn*_ was calculated in the same way. The synthetic sample was placed in the given bin. This process is repeated until the number of training subjects reaches 10 times of the original training dataset.

### Model Validation

To evaluate our proposed model, we utilized fivefold cross-validation for both classification and regression tasks. Specifically, we randomly divided the dataset into five portions. While one portion was used for testing, the remaining four portions were used as training data (70% for model training and 30% for model validation). This process was repeated five times until all portions of the dataset were treated as testing data. We evaluated the performance of risk prediction using accuracy, sensitivity, specificity, and the area under the receiver operating characteristic curve (AUC) across the five iterations. For cognitive score regression, we used Pearson’s correlation coefficient, MAE, and standard deviation of absolute error (STD of AE). The fivefold cross-validation experiment was repeated 50 times to reduce the variability and the 95% confidence interval was reported. All the experiments are performed on a Windows 10 workstation with Intel Xeon Silver 4116 CPU @ 2.10 GHz, 128 GB RAM, and dual GTX 1080ti GPUs.

### Most Discriminative Features Detection

In addition to the prediction of cognitive deficit, we seek to identify which brain regions contributed most to discriminate cognitive deficit. We used gradient-weighted class activation mapping (Grad-CAM) to highlight discriminative edges in the brain structural connectome map (Selvaraju et al., 2017). The Grad-CAM produces a coarse localization map highlighting predictive regions in the adjacency matrix by using gradient information of the last convolutional layer of the TL-CNN.

## Results

### Subjects

After excluding the data with large motion artifacts, we had a total of 80 very preterm infants out of 110 subjects in the final analysis. The 80 subjects had a mean (SD) gestational age at birth of 28.0 (2.4) weeks and postmenstrual age at the scan of 40.4 (0.6) weeks. There are 41 (51.3%) male subjects. The mean (SD) birth weight of the cohort was 1091.5 (385.3) g. We considered the infants with Bayley III cognitive scores <90 as a high-risk group (31 subjects) and with Bayley III cognitive scores ≥90 as a low-risk group (49 subjects) to develop later moderate/severe cognitive deficits (Spencer-Smith et al., 2015).

### Performance on Risk Stratification of Cognitive Deficits

**FIGURE 2:**
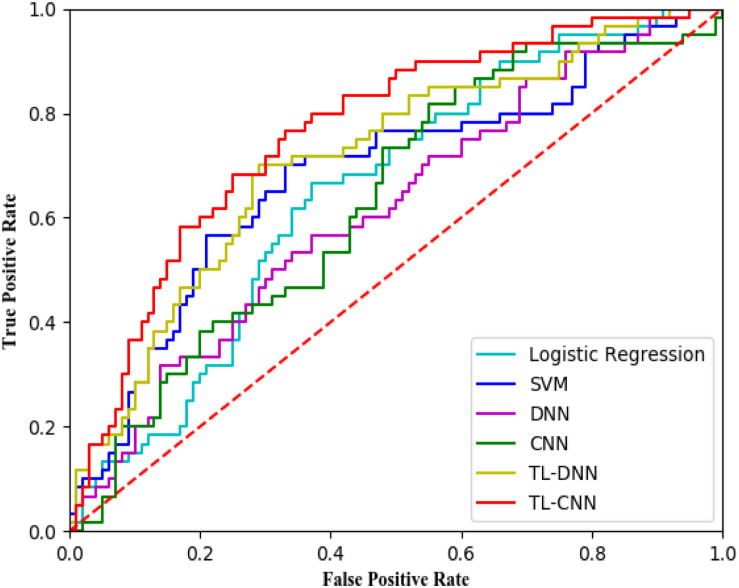
Receiver operating characteristic (ROC) curves of different prediction models using structural brain connectome at term-equivalent age in predicting cognitive deficits at 2 years corrected age in very preterm infants. The proposed TL-CNN model achieved the best area under the ROC curve among compared machine learning models. SVM, support vector machine; DNN, deep neural network; CNN, convolutional neural network; TL-DNN, transfer learning enhanced deep neural network; TL-CNN, transfer learning-enhanced convolutional neural network.

We compared the proposed TL-CNN model with LR, linear SVM, and TL-DNN in the identification of very preterm infants at high-risk for moderate/severe cognitive deficits (Table 1). The receiver operating characteristic curves of various machine learning models are displayed in Figure 2. Our proposed TL-CNN model achieved the best prediction performance among the compared models, with 74.5% on the balanced accuracy, 78.7% on specificity, 70.2% on sensitivity, and 0.75 on AUC. The CNN model achieved the lowest balanced accuracy of 67.3%, while DNN had the lowest AUC of 0.59. We also noted that the linear SVM model had better AUC than both DNN and CNN.

**TABLE 1:** Performance of various machine learning models in utilizing the structural connectome at term-equivalent age to predict cognitive deficits at 2 years corrected age in very preterm infants.

**Models**	**Balanced accuracy (%)**	**Specificity (%)**	**Sensitivity (%)**	**AUC**
LR	68.3 (67.5, 72.0)	72.3 (71.2, 73.8)	64.4 (62.4, 66.5)	0.65 (0.63, 0.67)
SVM	70.5 (67.7, 71.7)	76.9 (74.8, 78.9)	64.0 (61.8, 66.1)	0.69 (0.67, 0.71)
DNN	68.7 (65.7, 69.5)	75.0 (72.9, 77.1)	62.5 (60.4, 64.5)	0.59 (0.57, 0.61)
CNN	67.3 (66.2, 70.2)	73.7 (71.7, 75.6)	61.0 (59.1, 62.9)	0.64 (0.62, 0.73)
TL-DNN	71.6 (70.7, 73.1)	76.8 (75.8, 77.9)	66.4 (65.0, 67.7)	0.72 (0.70, 0.74)
**TL-CNN**	**74.5 (73.4, 76.0)**	**78.7 (77.2, 79.8)**	**70.2 (68.5, 70.7)**	**0.75 (0.74, 0.76)**

*Data in brackets are 95% confidence intervals. LR, logistic regression; SVM, support vector machine; TL-DNN, transfer learning enhanced deep neural network; TL-CNN, transfer learning enhanced convolutional neural network; AUC, area under the receiver operating characteristic curve.*

Without the TL strategy, the CNN model achieved better accuracy and AUC than DNN. A similar trend was observed on CNN and DNN models with the TL strategy. The TL-DNN achieved 71.6% on the balanced accuracy, 76.8% on specificity, 66.4% on sensitivity, and 0.72 on AUC. The proposed TL-CNN model significantly improved the cognitive deficit prediction over the TL-DNN model by 2.9% in accuracy (*p* = 0.005) and 3.0% in AUC (*p* = 0.008). This demonstrated the advantage of treating brain structural connectome as images instead of vectorized weights.

Transfer learning-enhanced models (i.e., TL-DNN and TL-CNN) had significantly better prediction performance than the models without TL (i.e., DNN and CNN). TL strategy significantly improved prediction accuracy and AUC of CNN by 7.2% (*p* < 0.001) and 11.6% (*p* < 0.001). Similarly, TL-DNN increased prediction accuracy and AUC of DNN by 2.9% (*p* = 0.002) and 3.5% (*p* < 0.001). These results illustrated the effectiveness of the TL approach in deep learning models on the prediction of cognitive deficit.

### Performance on the Prediction of Cognitive Scores

In the regression task, the proposed TL-CNN model had the highest Pearson’s correlation coefficient (*r* = 0.47, *p* < 0.001) between the predicted and actual cognitive scores compared to linear regression (*r* = 0.29, *p* < 0.001), support vector regression (SVR) (*r* = 0.32, *p* < 0.001), and TL-DNN (*r* = 0.37, *p* < 0.001) models (Table 2). TL-CNN had the lowest mean STD of AE of 9.5.

**TABLE 2:** Performance of various machine learning models in utilizing the structural connectome at term-equivalent age to predict Bayley-III cognitive scores at 2 years corrected age in very preterm infants.

**Models**	***r***	***p***	**MAE**	**STD of AE**
Linear regression	0.29 (0.27, 0.31)	<0.0001	20.1 (17.6, 22.6)	12.0 (10.7, 13.3)
SVR	0.32 (0.31, 0.34)	<0.0001	18.2 (15.1, 20.9)	11.4 (9.4, 13.4)
TL-DNN	0.37 (0.35, 0.39)	<0.0001	22.5 (20.0, 24.9)	11.2 (9.5, 13.0)
**TL-CNN**	**0.47 (0.45, 0.49)**	**<0.0001**	**16.2 (13.8, 18.5)**	**9.5 (7.8, 11.2)**

*Data in brackets are 95% confidence intervals. *r*, correlation between true and predicted Bayley-III cognitive scores; *p*, *p*-value (false discovery rate corrected) of one-sample *t*-test of *r*; MAE, mean absolute error; STD of AE, standard deviation of absolute error; SVR, support vector regression; TL-DNN, transfer learning enhanced deep neural network; TL-CNN, transfer learning enhanced convolutional neural network.*

### Discriminative Brain Structural Connectome

**FIGURE 3:**
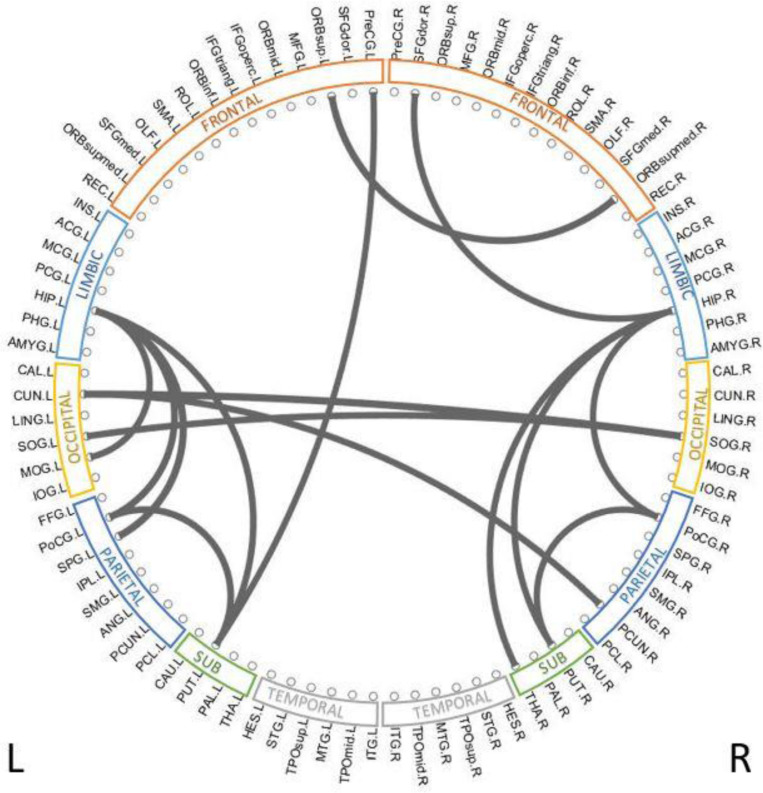
Top 15 discriminative brain structural connections identified by TL-CNN, a circos plot. The top three discriminative structural connections are located within frontal lobes, limbic lobes, and the subcortical structure.

To reveal which brain regions contributed to the prediction of cognitive deficits, we identified the predictive brain structural connections using the Grad-CAM method (Selvaraju et al., 2017). Table 3 displays the top 15 predictive brain structural connections. We further demonstrated the identified brain connections in a circos plot (Figure 3). The top three discriminative structural connections are located within frontal lobes, limbic lobes, and the subcortical structure. We also plotted those discriminative connections on a brain atlas region using BrainNet Viewer (Xia et al., 2013; Supplementary Figure 2).

**TABLE 3:** Top 15 discriminative brain structural connections for prediction of cognitive deficits.

**Brain region A**	**Abbreviation**	**Brain region B**	**Abbreviation**	***r***
**Top discriminative features**				
Precentral gyrus left	PreCG-L	Putamen left	PUT-L	0.39
Superior occipital gyrus left	SOG-L	Superior occipital gyrus right	SOG-R	0.37
Hippocampus left	HIP-L	Middle occipital gyrus left	MOG-L	0.34
Postcentral gyrus right	PoCG-R	Putamen right	PUT-R	0.33
Hippocampus right	HIP-R	Postcentral gyrus right	PoCG-R	0.33
Hippocampus left	HIP-L	Superior parietal gyrus left	SPG-L	0.33
Orbitofrontal cortex (superior) left	ORBsup-L	Orbitofrontal cortex (medial) right	ORBmed-R	0.29
Putamen left	PUT-L	Hippocampus left	HIP-L	0.28
Postcentral gyrus left	PoCG-L	Putamen left	PUT-L	0.27
Putamen right	PUT-R	Hippocampus right	HIP-R	−0.25
Postcentral gyrus left	PoCG-L	Hippocampus left	HIP-L	0.25
Hippocampus right	HIP-R	Thalamus right	THA-R	0.21
Cuneus left	CUN-L	Precuneus right	PCUN-R	−0.21
Cuneus left	CUN-L	Superior occipital gyrus right	SOG-R	0.20
Superior frontal gyrus (dorsal) right	SFGdor-R	Hippocampus right	HIP-R	0.19

*r, correlation between brain connectome weights and true Bayley-III cognitive scores.*

## Discussion

Early diagnosis and prediction of cognitive deficit for very preterm infants remain very challenging yet critical for early intervention. In this study, we proposed a TL-CNN model using brain structural connectome at term-equivalent age to predict future cognitive outcomes (i.e., standardized Bayley III cognitive scores). The TL-CNN model achieved promising performance in both risk classification and score regression tasks. For risk prediction of cognitive deficit, TL-CNN achieved a balanced accuracy of 74.5% and an AUC of 0.75. For regression of cognitive scores, the TL-CNN model had the best Pearson’s correlation coefficient among multiple machine learning models. These results demonstrated the feasibility and advantages of a deep learning model that may facilitate the early diagnosis and classification of cognitive deficit for very preterm infants at term-equivalent age.

The proposed TL-CNN model outperformed several peer machine learning models by using both spatial and topological locality information embedded in the adjacency matrix of the brain structural connectome. For those traditional and fully connected neuron-based DNN, the brain connectome is flattened to a vector (He et al., 2018; Chen et al., 2019). This approach discards important spatial and topological locality information from the connectome. By treating the brain structural connectome as 2D images, convolutional filters in CNN can inherently learn the spatial information. In this study, we adopted a neonatal AAL brain atlas (Shi et al., 2011). The regions in this atlas are numbered through 1–90 and spatially nearby regions have adjacent numbers. In the adjacency matrix of structural connectome, the location of the brain regions follows the original ordering of 1–90; therefore, brain regions near each other in the structural connectome are typically near each other in Euclidean/brain space. In this way, convolutional filters of CNN are able to learn spatial connectivity information of those “clustered” nearby regions. Meanwhile, those 2D grid convolutional filters move in both row and column directions across the whole adjacency matrix in a single convolutional layer. After a series of consecutive layers, the deep CNN model can integrate the topological locality information gradually. Thus, we believe that applying the deep CNN model on the adjacency matrix provides unique insight to learn latent spatial and topological locality embedded in the brain structural connectome. The significantly improved prediction performance by the proposed TL-CNN supports the rationale of our study design.

We applied CNN to learn the spatial and topological information of the structural connectome. In this study, we constructed the structural connectome based on a neonatal AAL brain atlas (Shi et al., 2011). The regions in this atlas are numbered through 1–90 and spatially nearby regions have adjacent numbers. Specifically, the neonatal AAL atlas arranged 90 brain regions into the following sections: frontal lobe (region: 1–28, 69–70), occipital lobe (region: 43–54), parietal lobe (region: 61–68), central structures (region: 55–60), and temporal lobe (region: 37–42, 71–90). Therefore, though not strictly speaking, brain regions near each other in the structural connectome are typically near each other in Euclidean/brain space. As CNN’s convolutional filters move across rows and columns of the structural connectome adjacency matrix in a moving-windows manner, the model was able to learn topological connectivity information. We tested the prediction performance with five different permuted connectome matrices. The TL-CNN achieved an accuracy of 68.8% (95% CI, 66.9, 70.7), and an AUC of 0.65 (95% CI, 0.63, 0.67), which was slightly lower than the performance of using original structure connectome matrix. This indicates that the order of the ROIs in the structural connectome matrix has an impact on the outcome prediction performance.

Transfer learning technique is essential for studies of very preterm infants using deep learning models. The big data revolution has boosted recent advances in deep learning techniques. Without large training samples, it is very difficult to train a complex deep learning model from scratch. Indeed, the linear SVM demonstrated better performance than deep learning models without the TL strategy in our study. Deep learning models trained with a small number of samples tend to be overfitted. Those relatively simple machine learning models (e.g., SVM) may achieve better performance. Unfortunately, the availability of annotated large brain imaging datasets with clinical and outcome information from very preterm infants is usually very limited, preventing the application of deep learning models in this research domain. The CNN model is a complex network consisting of millions of trainable weights that requires a large amount of data to update the weights when training the model. The TL technique addressed this issue by applying knowledge learned from a large dataset in the source domain to a new target task with limited data to improve performance and model robustness. In the present study, we transferred the knowledge (i.e., optimized weights) from a pre-trained model to the prediction/regression tasks in the target domain and then fine-tuned the model using brain structural connectome to optimize the performance of risk prediction/score regression. The increased performance supports our hypothesis regarding the effectiveness of the TL strategy.

The data balance and augmentation technique also improved the model training. Our dataset was imbalanced with a small number of subjects having low Bayley III cognitive scores. The imbalanced dataset may result in a model that is more likely to predict a high-risk subject into the majority low-risk group. Thus, we applied the data balance and augmentation technique before training any model in this work.

Identification of discriminative brain regions not only improves our understanding of the neurodevelopment of very preterm infants but also enhances the integrity of trained deep learning models. We applied the Grad-CAM method to rank the importance of individual links. Multiple brain regions such as postcentral gyrus, thalamus, and superior occipital gyrus were identified by our TL-CNN model to be predictive to cognitive deficits. These regions were also found to be predictive in our previous study using functional connectome on an independent cohort (He et al., 2018). In addition, postcentral gyrus, thalamus, and superior occipital gyrus were also reported in prior independent studies (Corbetta, 1998; Ouhaz et al., 2018), indicating their association with brain cognitive function. These somatosensory regions are thought to be part of the mirror system, which plays an important role in imitating, understanding, and learning for brain cognitive development (Acharya and Shukla, 2012). Furthermore, the identified most predictive regions have been associated with emotional regulation and memory (limbic lobe) (Catani et al., 2013), visual processing (occipital lobe) (Pöppel et al., 1978), and sensory, visual, and language information processing (parietal lobe) (Wolpert et al., 1998). Additionally, subcortical gray matter regions that play an important role in motion preparation and execution were also ranked highly by the proposed TL-CNN model (Chang et al., 2018).

We further performed a correlation analysis between the top 15 discriminative structural connectome connections and the cognitive outcomes at 2 years corrected age (Table 3). Briefly, the majority of brain connectome connections have a positive correlation with the cognitive scores. The increased connectivity strength of these connections would indicate a lower risk of cognitive deficits in very preterm infants at 2 years corrected age. This trend is consistent with our previous study (He and Parikh, 2016). In contrast, two brain connectome connections (Putamen right–Hippocampus right and Cuneus left–Precuneus right) are negatively correlated with cognitive scores, indicating that the increased connectivity strength of these two connections suggests a higher risk of cognitive deficits in very preterm infants at 2 years corrected age. Further investigation is required to unveil the underlying pathological mechanism of these brain connectome connections on brain cognitive functions.

There are several limitations to this study. First, we only internally validated our data in our cohort of very preterm infants. External datasets from independent studies or other research groups are necessary to externally validate the proposed TL-CNN models. Second, we only used brain structural connectome data for the outcome prediction. The integration of brain functional connectome and/or clinical data in our model is likely to improve prediction performance. Third, we constructed brain structural connectome based on an AAL brain atlas without cerebellum regions (Shi et al., 2011). However, the cerebellum regions have been conventionally recognized to have an impact on motor function and recently have been proven to associate with cognitive function (Schmahmann, 2019). The inclusion of the cerebellum regions when we construct the structural connectome may further enhance the prediction performance.

## Conclusion

In summary, this study proposed a deep learning model TL-CNN for early prediction of cognitive deficit in very preterm infants at 2 years corrected age using brain structural connectome derived from DTI obtained at term-equivalent age. The proposed model achieved improved performance by integrating multiple technique advances, including the convolution of CNN on adjacency matrix, TL strategy, and data balance and augmentation approach. The results suggest that deep learning models may facilitate early prediction of later neurodevelopmental outcomes in very preterm infants at term-equivalent age.

## Data Availability Statement

The raw data supporting the conclusions of this article will be made available by the authors, without undue reservation, to any qualified researcher.

## Ethics Statement

The studies involving human participants were reviewed and approved by the Institutional Review Board Nationwide Children’s Hospital. Written informed consent to participate in this study was provided by the participants’ legal guardian/next of kin.

## Author Contributions

MC, HL, NP, and LH designed the study. MC, HL, JW, and LH conducted the experiments and data analysis. All authors wrote the manuscript.

## Conflict of Interest

The authors declare that the research was conducted in the absence of any commercial or financial relationships that could be construed as a potential conflict of interest.
