# Supplementary material for: Early Prediction of Cognitive Deficit in Very Preterm Infants Using Brain Structural Connectome With Transfer Learning Enhanced Deep Convolutional Neural Networks
Source: Front Neurosci. 2020 Sep 18;14:858. doi: 10.3389/fnins.2020.00858 (PMC7530168; doi:10.3389/fnins.2020.00858)
Supplement: Supplementary file 1 [file Image_1.pdf]

Supplementary Material

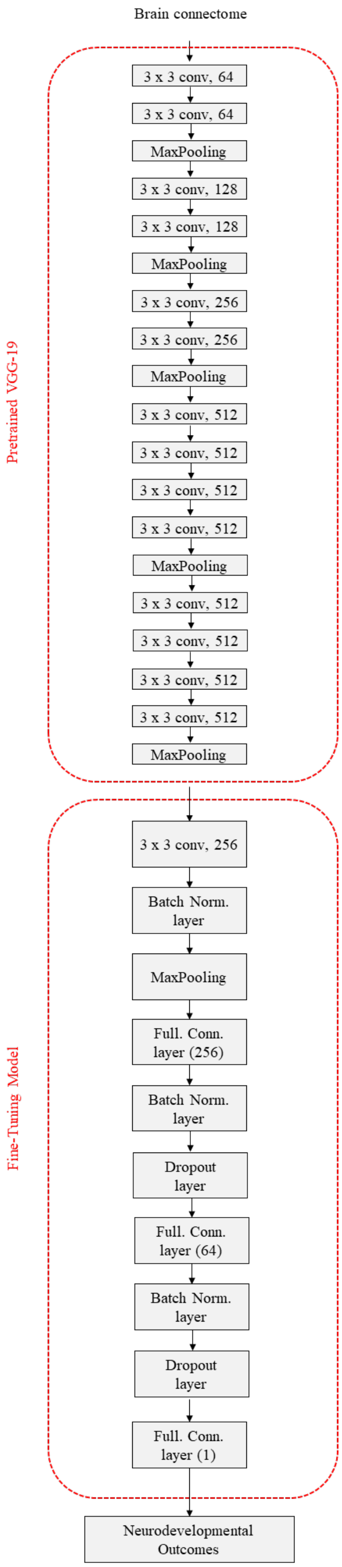

Supplemental Figure 1. Overview of the TL-enhanced CNN framework. The model is constructed convolutional layers, max-pooling layer, and fully connected layer.

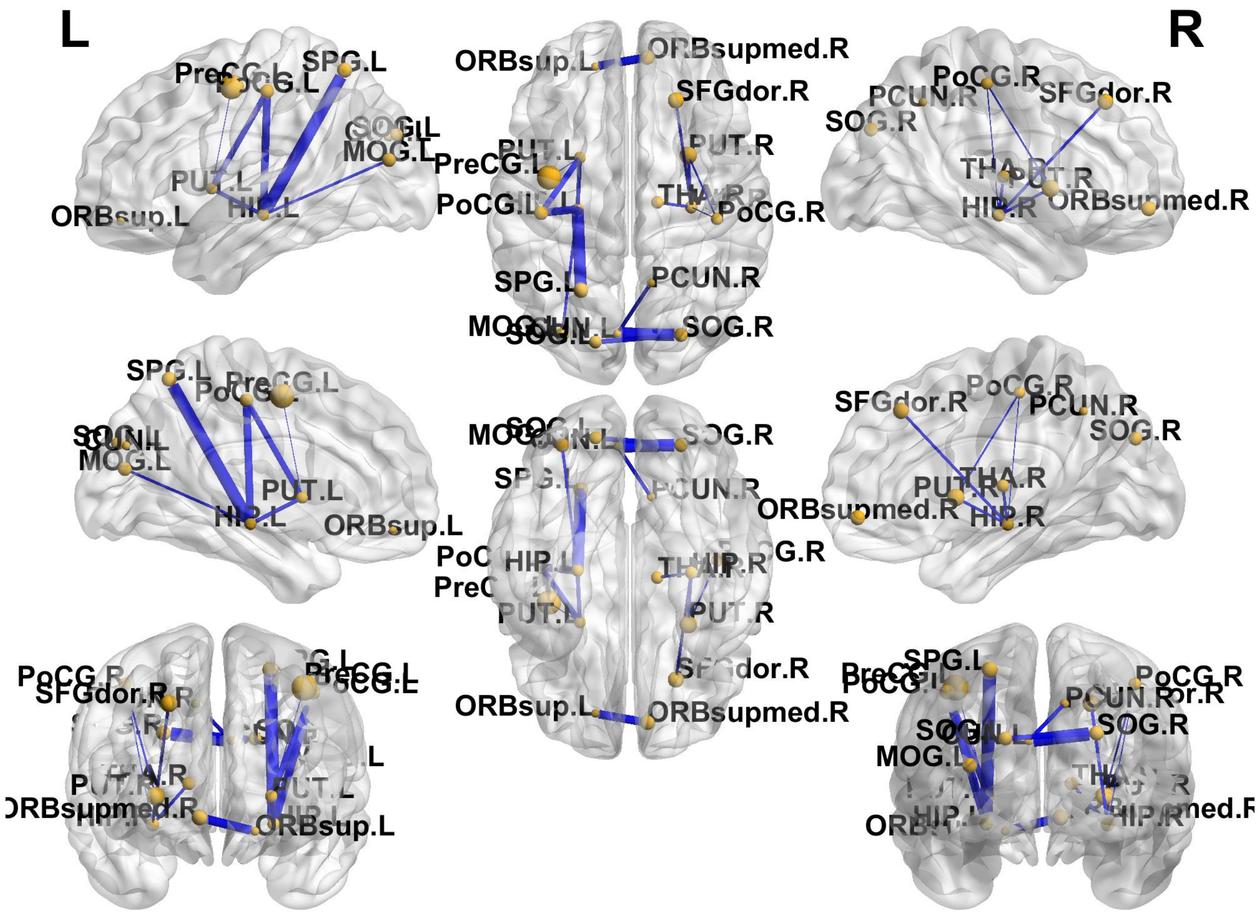

Supplemental Figure 2. Top 15 discriminative brain structural connections identified by the proposed CNN-TL model using the Grad-CAM method are shown on a standardized AAL brain atlas. The size of each node/region indicates the importance of that node/region in the prediction (i.e., more important regions are larger). The width of each segment (functional connection) indicates the predictive strength (i.e., more predictive regions are wider).
